# A network based covariance test for detecting multivariate eQTL in saccharomyces cerevisiae

**DOI:** 10.1186/s12918-015-0245-0

**Published:** 2016-01-11

**Authors:** Huili Yuan, Zhenye Li, Nelson L.S. Tang, Minghua Deng

**Affiliations:** LMAM, School of Mathematical Sciences, Peking University, Yiheyuan Road, Beijing, 100871 China; Department of Chemical Pathology, Prince of Wales Hospital, Faculty of Medicine, The Chinese University of Hong Kong, Shatin, Hong Kong, China; Center for Quantitative Biology, Peking University, Yiheyuan Road, Beijing, 100871 China; Center for Statistical Sciences, Peking University, Yiheyuan Road, Beijing, 100871 China

**Keywords:** eQTL, Pathway, Isoform

## Abstract

**Background:**

Expression quantitative trait locus (eQTL) analysis has been widely used to understand how genetic variations affect gene expressions in the biological systems. Traditional eQTL is investigated in a pair-wise manner in which one SNP affects the expression of one gene. In this way, some associated markers found in GWAS have been related to disease mechanism by eQTL study. However, in real life, biological process is usually performed by a group of genes. Although some methods have been proposed to identify a group of SNPs that affect the mean of gene expressions in the network, the change of co-expression pattern has not been considered. So we propose a process and algorithm to identify the marker which affects the co-expression pattern of a pathway. Considering two genes may have different correlations under different isoforms which is hard to detect by the linear test, we also consider the nonlinear test.

**Results:**

When we applied our method to yeast eQTL dataset profiled under both the glucose and ethanol conditions, we identified a total of 166 modules, with each module consisting of a group of genes and one eQTL where the eQTL regulate the co-expression patterns of the group of genes. We found that many of these modules have biological significance.

**Conclusions:**

We propose a network based covariance test to identify the SNP which affects the structure of a pathway. We also consider the nonlinear test as considering two genes may have different correlations under different isoforms which is hard to detect by linear test.

**Electronic supplementary material:**

The online version of this article (doi:10.1186/s12918-015-0245-0) contains supplementary material, which is available to authorized users.

## Background

GWAS aims to detect the association between genetic variation and complex diseases. Recent years, GWAS has found 2000 loci associated to complex diseases by statistical methods [1]. As the development of the next-generation sequencing and other high-throughput technology, various types of genome-scale datasets have been collected, providing opportunity to find the mechanism of genetic variation leading to complex diseases by connect the high-throughout data to GWAS. The eQTL study is one of them, which aims to uncover the genetic effects to gene expression and have been conducted in many organisms [2–5]. A common approach in eQTL data analysis is to consider association between each expression trait and each genetic marker through regression analysis. Despite great success with this approach, some regulatory signals may not be detected due to complex interaction between SNPs like epistasis.

Although most eQTL studies considered the expression levels of individual genes as response (single outcome variable), the change of correlation between genes under different genetic status still contains some biological information. For example, post-transcriptional regulations such as phosphorylations and dephosphorylations often affect the activities of transcriptional factors (TFs), which further affect the correlation among TF genes and TF target genes, also the co-expression patterns of the targets of TFs. However, such regulations are hard to be detected if only individual gene considered because there may be little change at the expression levels of individual TF genes. The approach considering “liquid association” (LA) between a pair of genes proposed by [6] is a method to identify such loci, which is later introduced into eQTL study [7]. Subsequently, conditional bi-variate normal model has been developed to capture the change of correlation between a pair of genes [8–10].

However, a biological process is usually performed by a group of genes (more than two genes as in the bi-variate model). Network approaches should be used to study these interactions [11–13]. If we want to see the effects of a cellular change to the organism, it is better for us to consider the change in a functional gene-set such as a pathway. Therefore, some papers has considered the multivariate circumstances by applying CCA to gene expressions and SNP (or CNV) data [14–16]. However, these methods do not consider the network structure when finding the association between gene sets and genetic variant, which will miss the information contained in the network. Li et al. [17], Kim and Xing [18], Zhang and Kim [19], Casale et al. [20] have considered pathway structure when studied the association between genetic variation and gene expression. However, they assume the network structure is the same (static) under different genetic variant. In fact, network structure may be dynamic and biologists have realized that differential network analysis will become a standard mode in network analysis and insightful discoveries could be made with differential network analysis [21]. For example, [22] identified a cancer point mutation in the kinase domain of RET, which causes multiple endocrine neoplasia type 2B by leading to a switch in peptide specificity and then altering the network structure.

So we propose a method to test whether the co-expression pattern in a pathway is affected by a SNP. Our goal is to test for a global change in covariance structure in each pathway, which is different from other network-based methods, which tries to detect non-zero edges from all pairs of genes. When we applied our method to a yeast eQTL dataset, we were able to find some pathway-SNP modules that have biological significance.

## Methods

**Fig. 1:**
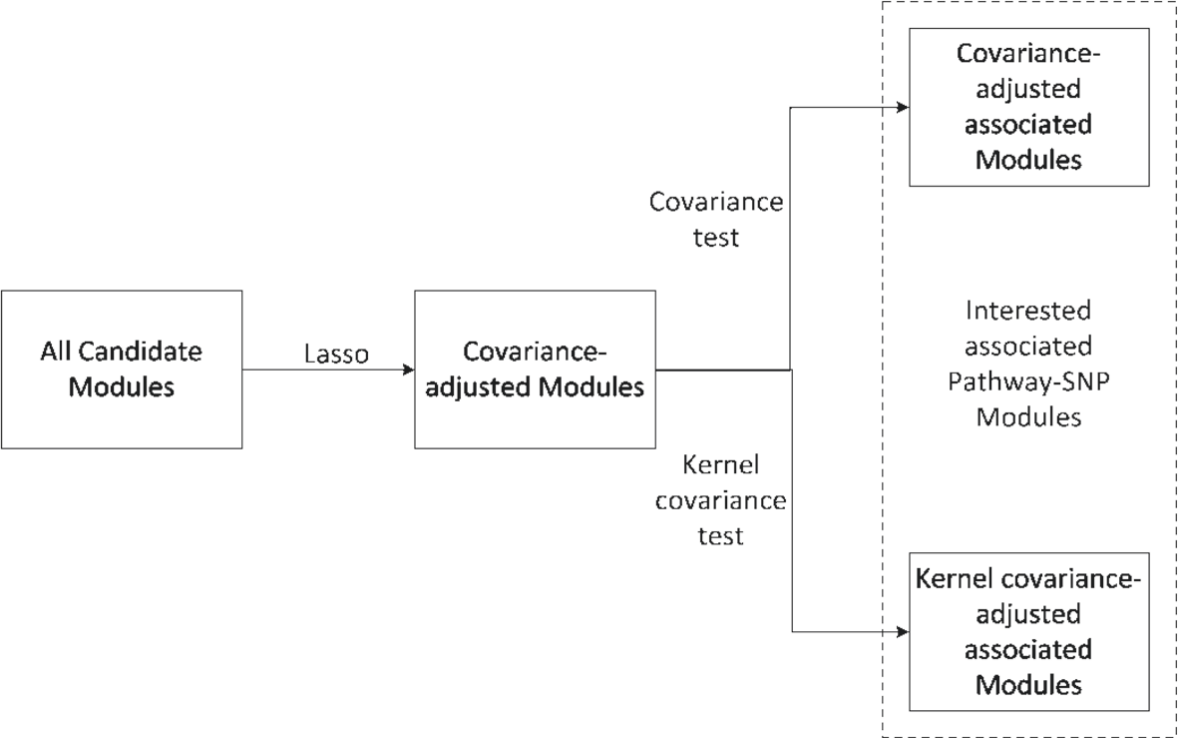
Flowchart of our strategy. Flowchart of our strategy for detecting pathway-associated SNP. We first perform Lasso to adjust the effects of SNPs on the means of gene expression. Then we use covariance test and kernel covariance test to select candidate pathway-SNP modules

Let (*X*_1_,*X*_2_,…,*X*_*p*_) be the expression levels of a group of genes and (*Z*_1_,*Z*_2_,…,*Z*_*m*_) be the set of SNPs. Suppose that there are n independent samples and let (*x*_1*i*_,*x*_2*i*_,…,*x*_*pi*_)_*i*=1,…,*n*_ denote the expression level of (*X*_1_,*X*_2_,…,*X*_*p*_) in the ith sample and (*Z*_1*i*_,*Z*_2*i*_,…,*Z*_*mi*_) denote the SNP types of the SNP set in the ith sample. Since the mean expression levels of (*X*_1_,*X*_2_,…,*X*_*p*_) are also possibly affected by some SNPs in (*Z*_1_,*Z*_2_,…,*Z*_*m*_), we can imitate the procedure in [9] that we first perform regression analysis or penalized regression analysis such as Lasso [23] or SCAD [24] to adjust the effects of (*Z*_1_,*Z*_2_,…,*Z*_*m*_) on the means and then model the residuals. We assume that the covariate-adjusted expression levels are appropriately centered to have mean values of zero and our interest is to test whether the covariate-adjusted covariance of expression levels is changed under each SNP. In our analysis, the group of genes are a pathway in KEGG [25]. Figure 1 describes our strategy to detect pathway-SNP associations. In this manuscript, we define a module as the collection of a SNP and a pathway, and our objective is to find pathway-SNP modules where the SNP affect the co-expression patterns among the genes in the pathway.

### Model

We use covariance test to find the pathway-SNP modules. There are three key elements of covariance test for a given gene set S. We consider the strategy similar to [26].

- **Calculation of T statistics.** We calculate a T statistics that reflects the difference of the covariance matrix of the two classes of samples. The statistics is calculated by estimating the Frobenius norm of the difference of the covariance matrix. We first perform the method by [27] to do the test:
  (1) $$ H_{0}: \Sigma_{1}=\Sigma_{2},\,\,\, H_{1}: \Sigma_{1}\neq\Sigma_{2}  $$
  where *Σ*_1_ is the covariance matrix of gene expression under one genotype and *Σ*_2_ is that of gene expression under the other genotype. Then we consider the nonlinear relationship between gene expressions by applying kernel method.
- **Estimation of significance level of T statistics.** We estimate the statistical significance (nominal P value) of the T statistics by using an empirical SNP-based permutation test procedure that preserves the complex correlation structure of the gene expression data. Specifically, we permute the SNP labels and recompute the T statistics of the gene set for the permuted data, which generates a null distribution for the T statistics. The empirical, nominal P value of the observed T statistics is then calculated relative to this null distribution. Importantly, the permutation of class labels preserves gene-gene correlations and, thus, provides a more biologically reasonable assessment of significance than would be obtained by permuting genes.
- **Adjustment for multiple hypothesis testing.** When an entire database of gene sets is evaluated, we adjust the estimated significance level to account for multiple hypothesis testing. We first normalize the T statistics for each gene set to account for the size of the set, yielding a normalized T statistics. We then control the proportion of false positives by calculating the false discovery rate (FDR) corresponding to each NT statistics. The FDR is the estimated probability that a set with a given NT statistics represents a false positive finding; it is computed by comparing the tails of the observed and null distributions for the NT statistics. To capture the change of the structure of the gene network, we consider the covariance of the gene expression.

### Test for high-dimensional covariance matrices

To simplify the problem, we just consider there are two possible values of each SNP. Covariance matrices under two genotypes of the SNP are denoted as *Σ*_1_ and *Σ*_2_, respectively. The primary interest is to test

$$H_{0}: \Sigma_{1} =\Sigma_{2},~~ H_{1}: \Sigma_{1}\neq\Sigma_{2} $$

which is a nontrivial statistical problem because the number of genes is greater than the number of samples sometimes. The test statistic for the hypothesis is formulated by targeting on tr (*Σ*_1_−*Σ*_2_)^2^, the squared Frobenius norm of *Σ*_1_−*Σ*_2_ [27]. Specifically, the test statistic is

$$T_{n_{1},n_{2}}=A_{n_{1}}+A_{n_{2}}-2C_{n_{1}n_{2}} $$

$${\small{\begin{aligned} A_{n_{h}}&=\frac{1}{n_{h}(n_{h}-1)}\sum\limits_{i\neq j}(X_{hi}'X_{hj})^{2}\\ &\quad-\frac{2}{n_{h}(n_{h}-1)(n_{h}-2)}\sum\limits_{i,j,k}^{*}X_{hi}'X_{hj}X_{hj}'X_{hk}\\ &\quad+\frac{1}{n_{h}(n_{h}-1)(n_{h}-2)(n_{h}-3)}\sum\limits_{i,j,k,l}^{*}X_{hi}'X_{hj}X_{hk}'X_{hl} \end{aligned}}} $$

$$\begin{aligned} C_{n_{1}n_{2}}&=\frac{1}{n_{1}(n_{2})}\sum\limits_{i}\sum\limits_{j}\left(X_{1i}'X_{2j}\right)^{2}\\ &\quad-\frac{1}{n_{1}n_{2}(n_{1}-1)}\sum\limits_{i,k}^{*}\sum\limits_{j}X_{1i}'X_{2j}X_{2j}'X_{1k}\\ &\quad-\frac{1}{n_{1}n_{2}(n_{2}-1)}\sum\limits_{i,k}^{*}\sum\limits_{j}X_{2i}'X_{1j}X_{1j}'X_{2k}\\ &\quad+\frac{1}{n_{1}n_{2}(n_{1}-1)(n_{2}-1)}\sum\limits_{i,k}^{*}\sum\limits_{j,l}^{*}X_{1i}'X_{2j}X_{1k}'X_{2l} \end{aligned} $$

where h refers to a subpopulation with a particular SNP.

For test *H*_0_:*Σ*_1_=*Σ*_2_=*Σ*_3_,*H*_1_:*Σ*_1_≠*Σ*_2_ or *Σ*_2_≠*Σ*_3_ We consider tr (*Σ*_1_−*Σ*_2_)^2^ + tr (*Σ*_2_−*Σ*_3_)^2^. Specifically, the test statistic is

$$T_{n_{1},n_{2}}+T_{n_{2},n_{3}} $$

where $T_{n_{2},n_{3}}$ is defined similar to $T_{n_{1},n_{2}}$.

### Kernel method

We generalize the method of [27] to the kernel space inspired by the method of [28]. We give the similar definition of Frobenius norm and covariance matrix. Let *p*_*x*_ and *p*_*y*_ be Borel probability measures defined on a domain *Ω*. Given observations *X*:={*x*_1_,…,*x*_*m*_} and *Y*:={*y*_1_,…,*y*_*n*_}, drawn independently and identically distributed(i.i.d.) from *p*_*x*_ and *p*_*y*_, respectively.

**Definition (HSDCC)** Given separable reproducing kernel Hilbert space (RKHS) $\mathcal {F}$, and measures *p*_*x*_,*p*_*y*_ over ($\mathcal {X},\Gamma $), we define the Hilbert-Schmidt Different Covariance Criterion(HSDCC) as the squared HS-norm of the difference of covariance *Σ*_*xx*_ and *Σ*_*yy*_:

$$HSDCC(p_{x},p_{y},\mathcal{F}):=\parallel\Sigma_{xx}-\Sigma_{yy}\parallel_{HS}^{2} $$

The detailed computation of above norm can be found in text of the Additional file 1. We give the unbiased statistics to $HSDCC(P_{x},P_{y},\mathcal {F})$ like [27]

$${\small{\begin{aligned} {}A_{n_{h}}&=\frac{1}{n_{h}(n_{h}-1)}\sum\limits_{i\neq j}k(X_{hi},X_{hj})^{2}\\ &\quad-\frac{2}{n_{h}(n_{h}-1)(n_{h}-2)}\sum\limits_{i,j,k}^{*}k(X_{hi},X_{hj})k(X_{hj},X_{hk})\\ &\quad+\!\frac{1}{n_{h}(n_{h}-1)(n_{h}-2)(n_{h}-3)}\!\sum\limits_{i,j,k,l}^{*}\!k(X_{hi},X_{hj})k(X_{hk},X_{hl}) \end{aligned}}} $$

$${\small{\begin{aligned} {}C_{n_{1}n_{2}}&=\frac{1}{n_{1}(n_{2})}\sum\limits_{i}\sum\limits_{j}k(X_{1i},X_{2j})^{2}\\ &\quad-\frac{1}{n_{1}n_{2}(n_{1}-1)}\sum\limits_{i,k}^{*}\sum\limits_{j}k(X_{1i},X_{2j})k(X_{2j},X_{1k})\\ &\quad-\frac{1}{n_{1}n_{2}(n_{2}-1)}\sum_{i,k}^{*}\sum\limits_{j}k(X_{2i},X_{1j})k(X_{1j},X_{2k})\\ &\quad+\frac{1}{n_{1}n_{2}(n_{1}-1)(n_{2}-1)}\sum\limits_{i,k}^{*}\sum\limits_{j,l}^{*}k(X_{1i},X_{2j})k(X_{1k},X_{2l}) \end{aligned}}} $$

$$T_{n_{1},n_{2}}=A_{n_{1}}+A_{n_{2}}-2C_{n_{1}n_{2}} $$

For test

*H*_0_:*Σ*_*xx*_=*Σ*_*yy*_=*Σ*_*zz*_,*H*_1_:*Σ*_*xx*_≠*Σ*_*yy*_ or *Σ*_*yy*_≠*Σ*_*zz*_

We consider $\parallel \Sigma _{\textit {xx}}-\Sigma _{\textit {yy}}\parallel _{\textit {HS}}^{2}+\parallel \Sigma _{\textit {yy}}-\Sigma _{\textit {zz}}\parallel _{\textit {HS}}^{2}$.

Specifically, the test statistic is

$$T_{n_{1},n_{2}}+T_{n_{2},n_{3}} $$

where $T_{n_{2},n_{3}}$ is defined similar to $T_{n_{1},n_{2}}$.

## Results

### Simulation

#### Comparison between linear method and kernel method

We performed a simulation study to evaluate the power of the proposed kernel methods, and compared the results with the primary method by [27]. Three models have been considered, as below.

Model 1: *X*_*ijk*_=*Z*_*ijk*_ + *θZ*_*ijk*+1_, where *Z*_*ijk*_ were i.i.d. standard normally distributed, and *θ*=0.5 in the null hypothesis while 0.2 or 0.3 in the alternative hypothesis.

Model 2: $\phantom {\dot {i}\!}X_{\textit {ijk}}=Z_{\textit {ijk}}^{3}+\theta Z_{ijk+1}^{3}$, where *Z*_*ijk*_ and *θ* were defined the same as that in Model 1.

Model 3: $\phantom {\dot {i}\!}X_{\textit {ijk}}=e^{Z_{\textit {ijk}}}+\theta e^{Z_{ijk+1}}$, where *Z*_*ijk*_ and *θ* were defined the same as that in Model 1.

The correlation between variables are linear in model 1, while the correlation between variables are nonlinear in model 2 and 3.

**Fig. 2:**
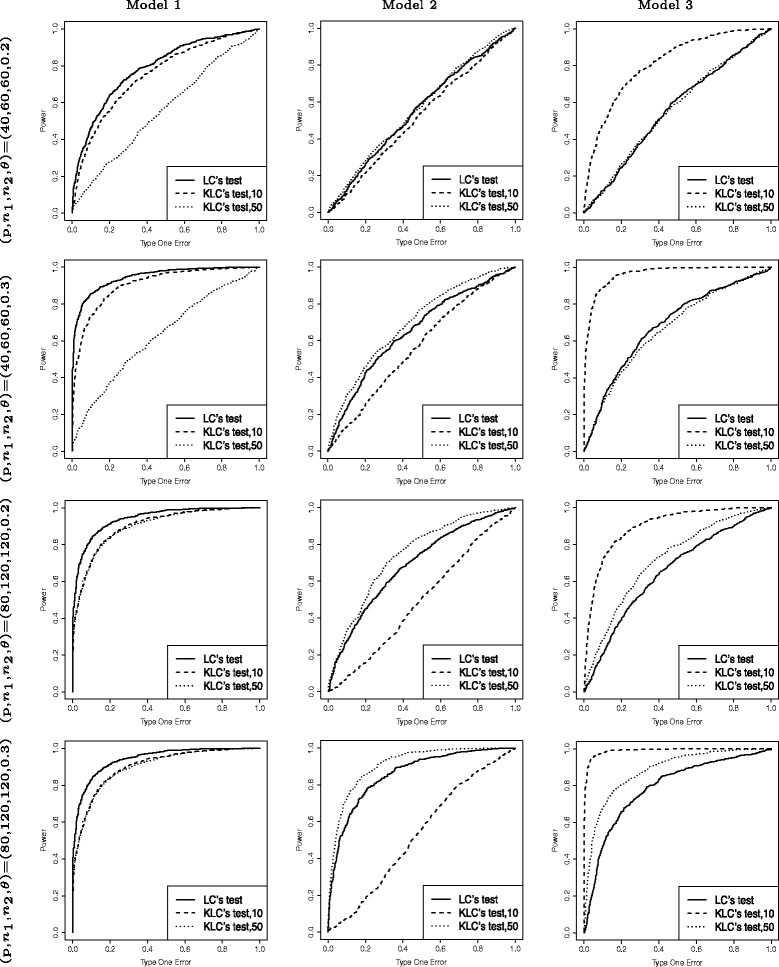
Comparison between linear method and kernel method. Simulations under different setups. Setup of the first column is under model 1, the second column is under model 2 and the third column is under model 3. First row: (p, *n*_1_,*n*_2_,*θ*) = (40, 60, 60, 0.2); Second row: (p, *n*_1_,*n*_2_,*θ*) = (40, 60, 60, 0.3); Third row: (p, *n*_1_,*n*_2_,*θ*) = (80, 120, 120, 0.2); Fourth row: (p, *n*_1_,*n*_2_,*θ*) = (80,120,120, 0.3)

We chose (p, *n*_1_,*n*_2_)=(40, 60, 60) and (80, 120, 120) respectively. The power of the tests are shown by ROC curves (Fig. 2). All the simulation results reported were based on 1000 simulations. We can see from the simulation that kernel methods with some parameters have higher power than the linear test when the true relationships between variables are nonlinear (Model 2 and Model 3). A similar simulation results with different setup of parameters can be found in Additional file 1: Figure S3.

#### Comparison between Chen et al.’s linear method and other method

We conducted a simulation to compare the power of Chen et al.’s method [27] and Tony Cai et al.’s method [29]. We consider four simulation setups represented different signal quantities and strength, the first of which is the same as the model 2 in [29].

Model 1: Let

$${}{\small{\begin{aligned} \Sigma^{*}&=(\sigma^{*}_{ij}), ~where~\omega^{*}_{ij}=0.5^{|i-j|}~for~1\leq i,j\leq p.\\ \Sigma\,&\,=\,D^{1/2}\Sigma^{*}D^{1/2},where~D\,=\,(d_{ij}),~d_{ii}\,=\,Unif(0.5,2.5),1\!\leq\! i\!\leq\! p\\ \Sigma_{1}&=\Sigma+\delta I, ~\Sigma_{2}=\Sigma+U+\delta I,~where~\delta\\ &=|\min\{\lambda_{min}(\Sigma+U),\lambda_{min}(\Sigma)\}|+0.05, \end{aligned}}} $$

*U*=(*u*_*kl*_) be a matrix with eight random nonzero entries, each with a magnitude generated from *Unif*(0,4)∗ max1≤*j*≤*pσ*_*jj*_. The number of each class samples is 50 and the number of variables is 50.

Model 2: *U*=(*u*_*kl*_) be a matrix with eight random nonzero entries, each with a magnitude generated from *Unif*(0,400)∗ max1≤*j*≤*pσ*_*jj*_.

Model 3: *U*=(*u*_*kl*_) be a matrix with 500 random nonzero entries, each with a magnitude generated from *Unif*(0,4)∗ max1≤*j*≤*pσ*_*jj*_.

Model 4: *U*=(*u*_*kl*_) be a matrix with 500 random nonzero entries, each with a magnitude generated from *Unif*(0,400)∗ max1≤*j*≤*pσ*_*jj*_.

**Fig. 3:**
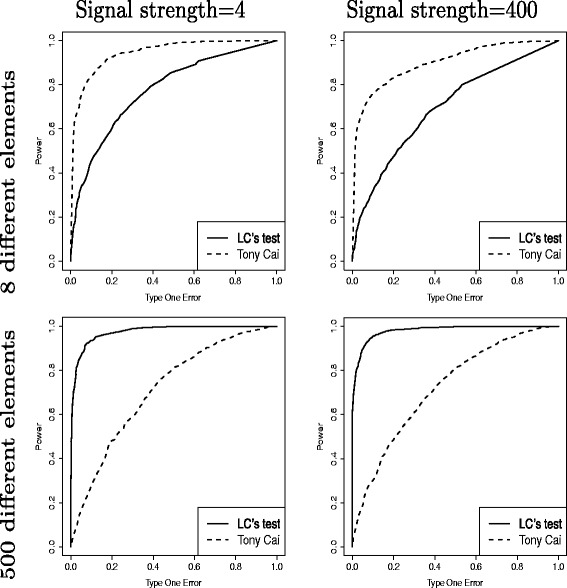
Comparison between Chen’s linear method and other method. *Topleft*: The two covariance matrices have eight different elements, each with a magnitude generated from *U* *n* *i* *f*(0,4)∗ max1≤*j*≤*p* *σ*_*jj*_; *Topright*: The two covariance matrices have eight different elements, each with a magnitude generated from *U* *n* *i* *f*(0,400)∗ max1≤*j*≤*p* *σ*_*jj*_; *Bottomleft*: The two covariance matrices have 500t different elements, each with a magnitude generated from *U* *n* *i* *f*(0,4)∗ max1≤*j*≤*p* *σ*_*jj*_; *Bottomright*: The two covariance matrices have 500 different elements, each with a magnitude generated from *U* *n* *i* *f*(0,400)∗ max1≤*j*≤*p* *σ*_*jj*_

As shown in Fig. 3, under the sparse setups (Model 1 and 2), the results of Tony Cai et al.’s method is much better than those of Chen et al.’s method. Chen et al.’s method is better than Tony Cai et al.’s method when the setups are not sparse (Model 3 and 4). Since Tony Cai et al.’s method corresponds to testing each element in the covariance matrix by Hoteling’s test and then give the judgement according to the maximum statistic of all of the Hoteling’s tests, so Chen et al.’s linear method has higher power than bi-variate model when the setups are not sparse. A similar simulation results with different number of samples can be found in Additional file 1: Figure S2.

### Real data results

#### Associated SNP and pathways

We analyzed the yeast dataset collected by Kruglyak and colleagues [30]. The expression data were downloaded from http://journals.plos.org/plosbiology/article?id=10.1371/journal.pbio.0060083, with 4482 genes measured in 109 segregants derived from a cross between BY and RM. The experiments were performed under two conditions, glucose and ethanol. We did the pre-processing like [10], after which 4419 genes and 820 merged markers remained. We mapped 4419 genes to 103 pathways and analyzed the effect of each SNP to each pathway. Therefore, we tested 103*820 times. The algorithm was implemented in R, which can be found at http://www.math.pku.edu.cn/teachers/dengmh/NetworkBiomarker.

**Fig. 4:**
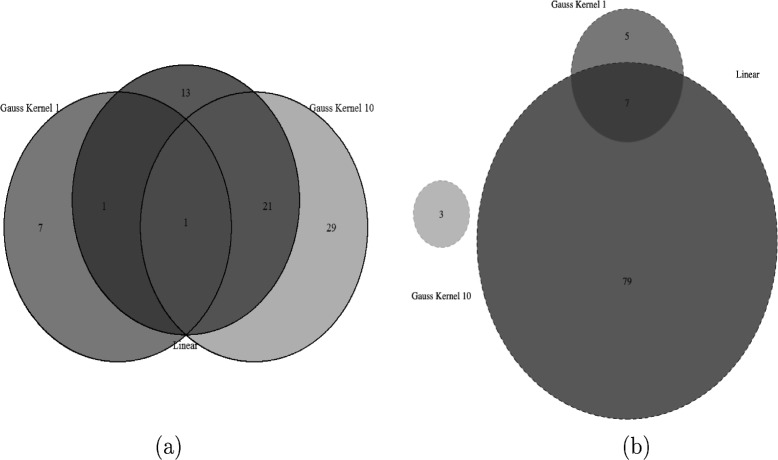
Different modules detected under different conditions by different method. **a** Detected modules under ethanol condition. We found 36 modules by covariance test, 9 modules by kernel covariance test with parameter 1 and 51 modules by kernel covariance test with parameter 10; **b** Detected modules under glucose condition. We found 86 modules by covariance test, 3 modules by kernel covariance test with parameter 10 and 12 modules by kernel covariance test with parameter 1

We performed covariance test and kernel covariance with parameter 1 and 10 respectively to the pathway-SNP adjusted modules. We consider 103 pathways in KEGG [25] (the number of genes in each pathway can be found in the Additional file 1: Figure S1) and 820 merged markers under ethanol and glucose condition respectively. We found 72 pathway-SNP modules under ethanol condition and 94 modules under glucose condition. Specifically, we found 36 modules by covariance test, 9 modules by kernel covariance test with parameter 1 and 51 modules by kernel covariance test with parameter 10 under ethanol condition, while 86 modules by covariance test, 3 modules by kernel covariance test with parameter 10 and 12 modules by kernel covariance test with parameter 1 under glucose condition (Fig. 4). Table 1 showed the associated pathways and SNPs under ethanol condition while Table 2 showed the associated pathways and SNPs under glucose condition.

**Table 1:** New associated pathways and SNPs under ethanol condition

Pathways	Associated markers
Glycolysis/Gluconeogenesis	*g* *O* *L*02^(10)^
Synthesis and degradation of ketone bodies	*Y* *L* *R*257*W* ^(10)^,*Y* *L* *R*261*C* ^(10)^
Steroid biosynthesis	*Y* *E* *L*021*W* ^*L*^, *Y* *F* *R*035*C* ^*L*^,
	*Y* *J* *L*001*W* ^*L*^, *Y* *J* *R*006*W* ^*L*,(10)^,
	*Y* *J* *L*007*C* ^*L*,(10)^,
Valine, leucine and isoleucine degradation	*Y* *O* *R*006*C* ^*L*^, *N* *O* *R*005*W* ^*L*,(10)^,
	*Y* *O* *R*051*C* ^*L*,(10)^, *Y* *O* *R*076*C* ^*L*,(10)^
Valine, leucine and isoleucine	*N* *L* *R*116*W* ^*L*,(10)^, *Y* *O* *R*076*C* ^*L*^,
biosynthesis	*Y* *C* *L*023*C* ^(10)^, *Y* *L* *R*257*W* ^(10)^
Histidine metabolism	*g* *O* *L*02^*L*,(10)^, *Y* *O* *R*025*W* ^(10)^
Tyrosine metabolism	*Y* *F* *L*019*C* ^*L*^, *g* *O* *L*02^*L*,(10)^
Phenylalanine metabolism	*g* *O* *L*02^*L*,(10)^
beta-Alanine metabolism	*g* *O* *L*02^*L*,(10)^
Taurine and hypotaurine metabolism	*g* *O* *L*02^(10)^
Selenocompound metabolism	*Y* *O* *R*006*C* ^*L*,(10)^, *Y* *O* *R*019*W* ^*L*,(10)^,
	*Y* *O* *R*025*W* ^*L*,(10)^, *N* *O* *R*005*W* ^*L*,(10)^
Purine metabolism	*N* *N* *L*035*W* ^(1)^
Cyanoamino acid metabolism	*Y* *L* *R*027*C* ^(1)^
Arachidonic acid metabolism	*g* *P* *L*09^(1)^
Linoleic acid metabolism	*Y* *F* *L*029*C* ^*L*,(1),(10)^, *Y* *F* *L*019*C* ^(1)^
Glyoxylate and dicarboxylate metabolism	*N* *N* *L*035*W* ^(1)^, *Y* *N* *L*074*C* ^(1)^
Porphyrin and chlorophyll metabolism	*N* *B* *R*008*W* ^(1)^
Sphingolipid metabolism	*Y* *H* *L*047*C* ^*L*^
Pantothenate and CoA biosynthesis	*Y* *G* *L*053*W* ^*L*^, *N* *L* *R*116*W* ^*L*,(10)^
Terpenoid backbone biosynthesis	*Y* *J* *L*007*C* ^*L*,(10)^, *Y* *J* *L*001*W* ^*L*,(10)^,
	*Y* *J* *R*006*W* ^*L*,(10)^, *N* *J* *R*006*C* ^*L*,(10)^
Sesquiterpenoid and triterpenoid	*Y* *O* *R*334*W* ^*L*^, *Y* *O* *R*343*C* ^*L*^,
biosynthesis	
	*Y* *L* *R*261*C* ^(10)^, *N* *L* *R*116*W* ^(10)^,
	*Y* *L* *R*257*W* ^(10)^
Metabolic pathways	*Y* *I* *L*078*W* ^(10)^, *Y* *L* *R*257*W* ^(10)^,
	*Y* *L* *R*308*W* ^(10)^, *N* *N* *L*035*W* ^(10)^,
	*g* *O* *L*02^(10)^, *Y* *O* *R*006*C* ^(10)^,
	*Y* *O* *R*051*C* ^(10)^,*Y* *O* *R*019*W* ^(10)^,
	*Y* *N* *L*066*W* ^(10)^,*Y* *L* *R*261*C* ^(10)^
Biosynthesis of secondary metabolites	*Y* *O* *R*025*W* ^(10)^, *Y* *O* *R*063*W* ^(10)^
Carbon metabolism	*Y* *O* *R*019*W* ^(10)^
2-Oxocarboxylic acid metabolism	*Y* *L* *R*261*C* ^*L*,(10)^, *Y* *L* *R*308*W* ^*L*,(10)^,
	*Y* *C* *L*022*C* ^(10)^, *Y* *L* *R*265*C* ^(10)^,
	*N* *L* *R*116*W* ^(10)^,*Y* *L* *R*322*W* ^*L*^
mRNA surveillance pathway	*Y* *O* *R*072*W* ^*L*^
Mismatch repair	*g* *K* *R*08^*L*^
Non-homologous end	*Y* *G* *R*006*W* ^*L*^
Biosynthesis of amino acids	*g* *O* *L*02^(10)^
MAPK signaling pathway	*Y* *D* *R*164*C* ^(10)^, *g* *D* *R*10^(10)^

L means detected by covariance test, (1) means detected by kernel covariance test with parameter 1 and (10) means detected by kernel covariance test with parameter 10. The FDR of the covariance test, kernel covariance test with parameter 1 and kernel covariance test with parameter 10 are 0.25, 0.33 and 0.25 respectively. The FWER of the test by Tony Cai is 0.2

**Table 2:** New associated pathways and SNPs under glucose condition

Pathways	Associated markers
Synthesis and degradation of ketone bodies	*g* *J* *L*07^(10)^
Inositol phosphate metabolism	*Y* *B* *R*259*W* ^(10)^
Riboflavin metabolism	*Y* *M* *L*056*C* ^(10)^
Fatty acid degradation	*Y* *B* *R*045*C* ^(1)^
Cysteine and methionine metabolism	*Y* *G* *L*195*W* ^(1)^
Valine, leucine and isoleucine biosynthesis	*Y* *C* *L*025*C* ^*L*^,*N* *G* *R*093*C* ^*L*^
	*Y* *O* *R*253*W* ^*L*^,*Y* *O* *R*274*W* ^*L*^
	*Y* *O* *R*326*W* ^*L*^,*Y* *O* *R*334*W* ^*L*^
	*Y* *O* *R*343*C* ^*L*^,*Y* *C* *L*022*C* ^(1)^
Phenylalanine metabolism	*Y* *J* *R*040*W* ^*L*^,*Y* *O* *L*123*W* ^*L*^
	*Y* *O* *L*118*C* ^*L*^,*Y* *O* *L*106*W* ^*L*^
	*Y* *O* *L*093*W* ^*L*^,*Y* *O* *L*088*C* ^*L*^
	*g* *O* *L*02^*L*,(1)^
beta-Alanine metabolism	*Y* *B* *R*271*W* ^*L*^,*g* *O* *L*02^*L*,(1)^
	*N* *J* *R*007*C* ^*L*^,*Y* *O* *L*106*W* ^*L*^
Arachidonic acid metabolism	*Y* *I* *R*022*W* ^*L*,(1)^
Vitamin B6 metabolism	*Y* *K* *L*118*W* ^(1)^
Porphyrin and chlorophyll metabolism	*Y* *M* *L*071*C* ^(1)^,*g* *F* *L*02^*L*^
Degradation of aromatic compounds	*Y* *M* *R*316*C* ^*L*,(1)^,*Y* *M* *R*316*C* ^*L*^
ABC transporters	*Y* *B* *R*131*W* ^*L*,(1)^,*Y* *B* *R*137*W* ^*L*^
Glycolysis/Gluconeogenesis	*Y* *J* *R*071*W* ^*L*^
Pentose phosphate pathway	*N* *O* *L*043*W* ^*L*^, *Y* *O* *L*151*W* ^*L*^
	*Y* *O* *L*123*W* ^*L*^, *Y* *O* *L*094*C* ^*L*^
	*Y* *O* *L*093*W* ^*L*^, *Y* *O* *L*088*C* ^*L*^
	*g* *O* *L*02^*L*^
Pentose and glucuronate interconversions	*Y* *G* *L*263*W* ^*L*^
Purine metabolism	*Y* *L* *R*140*W* ^*L*^
Pyrimidine metabolism	*Y* *B* *L*010*C* ^*L*^, *Y* *G* *L*217*C* ^*L*^
Glycine, serine and threonine metabolism	*Y* *C* *L*065*W* ^*L*^, *Y* *J* *R*038*C* ^*L*^
Lysine biosynthesis	*Y* *B* *R*087*W* ^*L*^
Histidine metabolism	*Y* *B* *R*271*W* ^*L*^, *N* *J* *R*007*C* ^*L*^
	*Y* *J* *R*040*W* ^*L*^, *Y* *J* *R*057*W* ^*L*^
	*Y* *O* *L*106*W* ^*L*^, *Y* *O* *L*093*W* ^*L*^,
	*g* *O* *L*02^*L*,(1)^
Tyrosine metabolism	*Y* *O* *L*123*W* ^*L*^, *Y* *O* *L*106*W* ^*L*^
	*Y* *O* *L*094*C* ^*L*^, *g* *O* *L*02^*L*,(1)^
Cyanoamino acid metabolism	*Y* *D* *R*351*W* ^*L*^
Starch and sucrose metabolism	*Y* *E* *R*095*W* ^*L*^,*Y* *E* *R*116*C* ^*L*^
Linoleic acid metabolism	*N* *D* *R*174*C* ^*L*^
Butanoate metabolism	*Y* *B* *R*271*W* ^*L*^
Pantothenate and CoA biosynthesis	*Y* *O* *R*274*W* ^*L*^
Lipoic acid metabolism	*g* *L* *L*01^*L*^,*Y* *N* *L*158*W* ^*L*^
Folate biosynthesis	*N* *M* *L*013*W* ^*L*^,*Y* *N* *L*066*W* ^*L*^,*Y* *N* *L*050*C* ^*L*^
Sesquiterpenoid and triterpenoid biosynthesis	*Y* *M* *R*084*W* ^*L*^
Aminoacyl-tRNA biosynthesis	*Y* *C* *L*065*W* ^*L*^,*Y* *C* *L*047*C* ^*L*^
	*Y* *C* *L*039*W* ^*L*^,*N* *J* *R*007*C* ^*L*^,*Y* *N* *L*010*W* ^*L*^
Biosynthesis of unsaturated fatty acids	*Y* *F* *L*029*C* ^*L*^
Metabolic pathways	*Y* *C* *L*065*W* ^*L*^,*Y* *J* *R*071*W* ^*L*^
Biosynthesis of secondary metabolites	*Y* *J* *R*038*C*6*L*
Biosynthesis of amino acids	*Y* *J* *R*071*W* ^*L*^,*Y* *O* *L*123*W* ^*L*^
	*Y* *O* *L*118*C* ^*L*^,*Y* *O* *L*106*W* ^*L*^
	*Y* *O* *L*094*C* ^*L*^,*Y* *O* *L*093*W* ^*L*^
	*Y* *O* *L*088*C* ^*L*^,*g* *O* *L*02^*L*^
Ribosome	*Y* *A* *R*035*W* ^*L*^,*Y* *J* *L*026*W* ^*L*^
RNA transport	*Y* *B* *L*010*C* ^*L*^
RNA polymerase	*Y* *L* *R*140*W* ^*L*^
Proteasome	*Y* *B* *L*010*C* ^*L*^
Phosphatidylinositol signaling system	*Y* *B* *R*045*C* ^*L*^
Meiosis - yeast	*Y* *O* *L*106*W* ^*L*^

L means detected by covariance test, (1) means detected by kernel covariance test with parameter 1 and (10) means detected by kernel covariance test with parameter 10. The FDR of the covariance test, kernel covariance test with parameter 1 and kernel covariance test with parameter 10 are 0.20, 0.24 and 0.33 respectively. The FWER of the test by Tony Cai is 0.2

#### Kernel Method found isoform-specific structure change

**Fig. 5:**
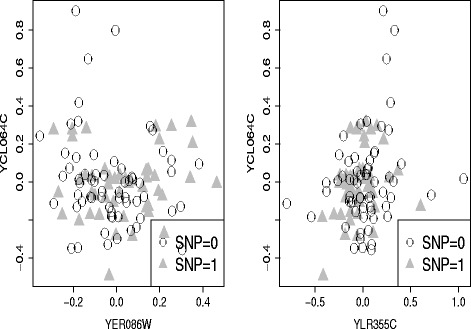
Isoform-specific structure change. *Left*: Scatter plot of gene YER086W and YCL064C under two different genotypes of YCL023C; *Right*: Scatter plot of gene YLR355C and YCL064C under two different genotypes of YCL023C. We found the associated pathway-SNP modules only by kernel covariance test. The scatter figures show that YER086W-YCL064C and YLR355C-YCL064C were nonlinear correlated under genotypes of marker YCL023C

In our result, we found Valine, leucine and isoleucine biosynthesis pathway was associated with YCL023C marker only by kernel method under ethanol condition. Figure 5 shows the non-linear correlation between two pairs of genes, YER086W-YCL064C and YLR355C-YCL064C were nonlinear correlated with genotypes of YCL023C. And more than 10 isoforms of YER086W and 6 isoforms of YLR355C have been found (Saccharomyces Genome Database, http://www.yeastgenome.org/). The nonlinear correlation between two pairs of genes might be caused by samples in different isoforms. Specifically, two genes may be positive correlated under one isoform while negetive correlated under another isoform. However, the correlation of two genes might be missed if when we only considered linear correlation.

#### Linoleic acid metabolism is associated to cell cycle

**Fig. 6:**
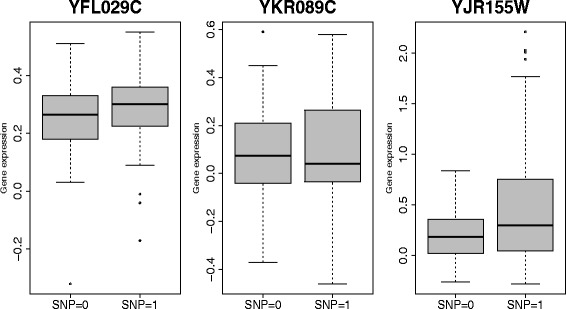
Failure to detect correlation between single gene expression level and genotype of YFL029C. *Left*: Boxplot of expression level of YFL029C; *middle*, Boxplot of expression level of YKR089C; *right*: Boxplot of expression level of YJR155W. We can see that the means of YKR089C and YJR155W expressions do not change significantly

**Fig. 7:**
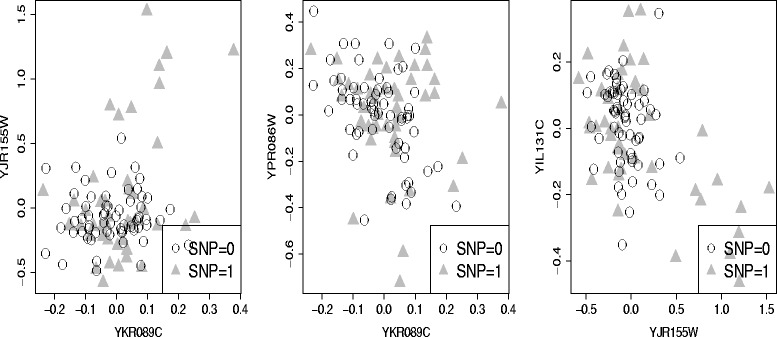
Three examples of differential coexpression patterns of 2 genes due to genotype of YFL029C. *Left*: the co-expression patterns between the two genes YKR089C and YJR155W depend on the genotype of YFL029C. For samples with genotype 1, the co-expression correlation is different from the other samples. *Middle*: the co-expression patterns between the two genes YKR089C and YPR086W depend on the genotype of YFL029C. *Right*: the co-expression patterns between the two genes YJR155W and YIL131C depend on the genotype of YFL029C

**Fig. 8:**
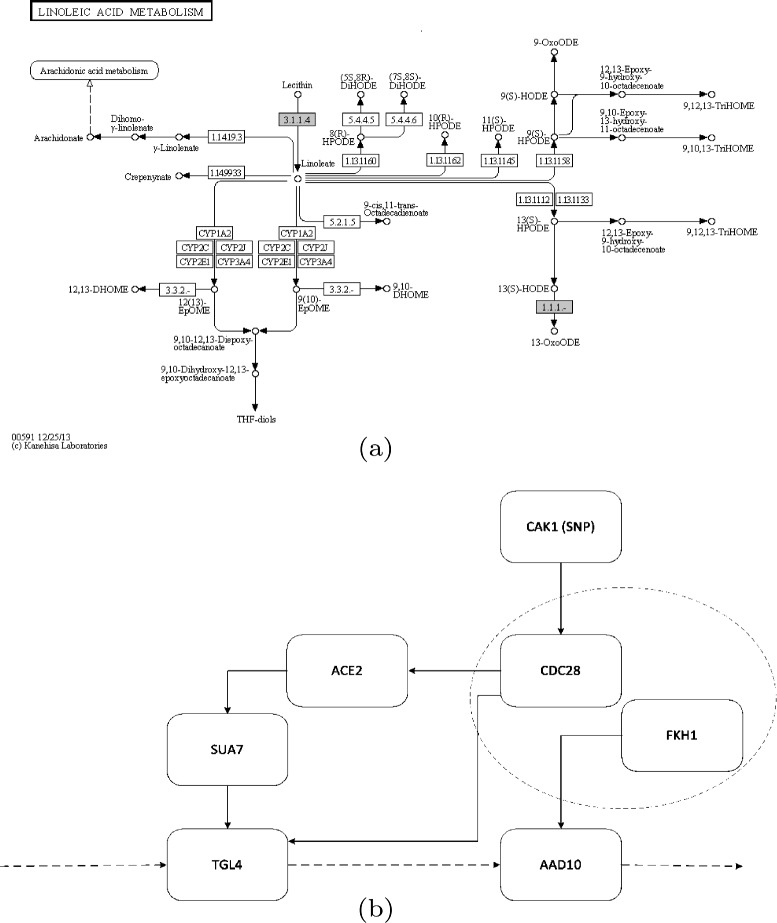
The possible regulatory mechanism of the marker YFL029C to Linoleic acid metabolism pathway. **a** KEGG pathway of Linoleic acid metabolism pathway; **b** The potential regulatory relationship between marker YFL029C and Linoleic acid metabolism pathway

Our method found YFL029C is associated with linoleic acid metabolism pathway under ethanol condition. With single gene correlation analysis, both of the mean of expression levels of YKR089C (TGL4) and YJR155W (AAD10) were not associated with YFL029C (Fig. 6 middle and right). Specifically, with p-value 0.5174 and 0.002804 (not significant for multiple test). However, the scatterplot after correction shows the correlation of two genes change apparently under YFL029C (Fig. 7 left). Under one status of SNP, the two genes are positive correlated while under the other status of SNP, the two are nearly independent. To understand this from the biological meaning which was showned in Fig. 8b, we found that marker locates in gene CAK1 (The expression of CAK1 is slightly different under two SNP status which was shown in Fig. 6 left.), the product of which can increase the activity of CDC28 [31]. CDC28 plays an important role in cell cycle. It can control the progress of cell cycle by phosphorylate different transcription factor. In our case, CDC28 phosphorylate ACE2 [32] which can increase the activity of transcription factor, SUA7 [33]. SUA7 is the transcription factor of TGL4, which is a lipase in linoleic acid metabolism pathway. Meanwhile, CDC28 and FKH1 can form complex [34] and FKH1 is the transcription factor of AAD10, which is another enzyme in linoleic acid metabolism pathway. The correlation between YKR089C and its TF was shown in Fig. 7 middle and the correlation between YJR155W and its TF was shown in Fig. 7 right. From the structure of the pathway in KEGG [25] as shown in Fig. 8a, the different status of the SNP YFL029C might lead to different amounts of intermediate product in the pathway.

## Discussion and conclusion

We propose a network based covariance test to identify the marker which affects the structure of a pathway. It has an advantage that a static network structure is not assumed. The biomarker we defined is the SNP associated to the structure of genes in the pathway. Considering two genes may have different correlations under different isoforms which is hard to detect by linear test, so we also consider the nonlinear test. We identified a total of 166 modules, with each module consisting of a group of genes and one eQTL where the eQTL regulate the co-expression patterns of the group of genes. We found that many of these modules have biological interpretations. Till now, we consider the difference of two networks by covariance matrix and covariance operators. We will focus on difference of precision matrix in the future research.

## Additional file

Additional file 1 **Supplementary materials for the computation of HSDCC and additional figures Figures S1–S3.** (PDF 1474 kb)
