# Supplementary material for: A network based covariance test for detecting multivariate eQTL in saccharomyces cerevisiae
Source: BMC Syst Biol. 2016 Jan 11;10(Suppl 1):8. doi: 10.1186/s12918-015-0245-0 (PMC4895706; doi:10.1186/s12918-015-0245-0)
Supplement: Supplementary file 1 — Supplementary materials for the computation of HSDCC and additional figures Figures S1–S3. (PDF 1474 kb) [file 12918_2015_245_MOESM1_ESM.pdf]

RESEARCH

Supplementary materials for 'A network based covariance test for detecting multivariate eQTL in saccharomyces cerevisiae'

Huili Yuan[1], Zhenye Li[1], Nelson L. S. Tang[2] and Minghua Deng[1,3,4*]

*Correspondence:
dengmh@math.pku.edu.cn
[1]LMAM, School of Mathematical Sciences, Peking University, Yiheyuan Road, 100871 Beijing, China
Full list of author information is available at the end of the article

1 Supporting Information

1.1 Preliminary[1]

Now consider a Hilbert space $\mathcal{F}$ of functions from R^p to R. Then $\mathcal{F}$ is a reproducing kernel Hilbert space(RKHS) if for each x $\in R^p$, the Dirac evaluation operator δ_x :$\mathcal{F}\rightarrow$ R, which maps $f \in \mathcal{F}$ to f(x) $\in$ R, is a bounded linear functional. To each point x $\in R^p$, there corresponds an element ϕ(x) $\in \mathcal{F}$ such that $\langle\phi(x),\phi(x^{'})\rangle_{\mathcal{F}}=k(\text{x},x^{'})$, where k : $R^p \times R^p \rightarrow$R is a unique positive definite kernel.

Hilbert-Schmidt Norm. Denote by $C:\mathcal{G}\rightarrow\mathcal{F}$ a linear operator. Then provided the sum converges, the Hilbert-Schmidt(HS) norm of C is defined as

$$\parallel C \parallel^2_{HS}:=\sum_{i,j}\langle C\nu_i,\mu_j\rangle^2_{\mathcal{F}} \tag{1}$$

where ν_i and μ_j are orthonormal bases of $\mathcal{F}$ and $\mathcal{G}$ respectively. It is easy to see that this generalises the Frobenius norm on matrices.

Hilbert-Schmidt Operator. A linear operator $C:\mathcal{G}\rightarrow\mathcal{F}$ is called a Hilbert-Schmidt operator if its HS norm exists. The set of Hilbert-Schmidt operators HS($\mathcal{G}$,$\mathcal{F}$): $\mathcal{G}\rightarrow\mathcal{F}$ is a separable Hilbert space with inner product

$$\langle C,D\rangle_{HS}:=\sum_{i,j}\langle C\nu_i,\mu_j\rangle_{\mathcal{F}}\langle D\nu_i,\mu_j\rangle_{\mathcal{F}}$$

Tensor Product. Let $f\in\mathcal{F}$ and $g\in\mathcal{G}$. Then the tensor product operator $f\otimes g$: $\mathcal{G}\rightarrow\mathcal{F}$ is defined as

$$(f\otimes g)h:=f\langle g,h\rangle_{\mathcal{G}} \ for\ all\ h\in\mathcal{G} \tag{2}$$

Moreover, by the definition of the HS norm, we can compute the HS norm of $f\otimes g$ via

$$\parallel f\otimes g\parallel^2_{HS}=\langle f\otimes g,f\otimes g\rangle_{HS}=\langle f,(f\otimes g)g\rangle_{\mathcal{F}}=\parallel f\parallel^2_{\mathcal{F}}\parallel g\parallel^2_{\mathcal{G}} \tag{3}$$

Mean.

$$\langle\mu_x,f\rangle_{\mathcal{F}}:=E_x[\langle\phi(x),f\rangle_{\mathcal{F}}]=E_x[f(x)], \tag{4}$$
$$\langle\mu_y,g\rangle_{\mathcal{G}}:=E_y[\langle\psi(y),g\rangle_{\mathcal{G}}]=E_y[g(y)], \tag{5}$$

where ϕ is the feature map from $\mathcal{X}$ to the RKHS $\mathcal{F}$, and ψ maps from $\mathcal{Y}$ to $\mathcal{G}$. Finally, $\parallel \mu_x \parallel_{\mathcal{F}}^2$ can be computed by applying the expectation twice via

$$\parallel \mu_x \parallel_{\mathcal{F}}^2 = E_{x,x'}[\langle \phi(x), \phi(x') \rangle_{\mathcal{F}}] = E_{x,x'}[k(x,x')] \tag{6}$$

Here the expectation is taken over independent copies x,x' taken from p_x.

Covariance Operator. The covariance operator associated with the joint measure p_x on ($\mathcal{X}$,Γ) is a linear operator $\Sigma_{xx} : \mathcal{F} \rightarrow \mathcal{F}$ defined as

$$\Sigma_{xx} := E_x[(\phi(x) - \mu_x) \otimes (\phi(x) - \mu_x)] = \underbrace{E_x[\phi(x) \otimes \phi(x)]}_{:=\widetilde{\Sigma}_{xx}} - \underbrace{\mu_x \otimes \mu_x}_{M_{xx}} \tag{7}$$

Similarly, $\Sigma_{yy} : \mathcal{G} \rightarrow \mathcal{G}$ is defined as

$$\Sigma_{yy} := E_y[(\psi(y) - \mu_y) \otimes (\psi(y) - \mu_y)] = \underbrace{E_y[\psi(y) \otimes \psi(y)]}_{:=\widetilde{\Sigma}_{yy}} - \underbrace{\mu_y \otimes \mu_y}_{M_{yy}} \tag{8}$$

1.2 Hilbert -Schmidt Different Covariance Criterion

Now, we assume that $\mathcal{X} = \mathcal{Y}$,$\Gamma = \Lambda$, so $\phi = \psi$

Defition(HSDCC).Given separable RKHSs $\mathcal{F}$, $\mathcal{G}$ and joint measures p_x, p_y over ($\mathcal{X}$,Γ) and ($\mathcal{Y}$,Λ),we define the Hilbert-Schmidt Different Covariance Criterion(HSDCC) as the squared HS-norm of the difference of covariance Σ_{xx} and Σ_{yy}:

$$HSDCC(p_x, p_y, \mathcal{F}) :=\parallel \Sigma_{xx} - \Sigma_{yy} \parallel_{HS}^2 \tag{9}$$

To compute it we need to express HSDCC in terms of kernel functions. This is achieved by the following lemma:

Lemma 1 (HSDCC in terms of kernels).

$$\begin{aligned} HSDCC(P_x, P_y, \mathcal{F}) =& E_{x,x'}k(x,x')^2 - 2E_{x'}[E_x k(x,x')E_{x''}k(x',x'')] + (E_{x,x'}k(x,x'))^2 \\ &+ E_{y,y'}k(y,y')^2 - 2E_{y'}[E_y k(y,y')E_{y''}k(y',y'')] + (E_{y,y'}k(y,y'))^2 \\ &- 2E_{x,y'}k(x,y')^2 + 2E_{y'}[E_x k(x,y')E_{x'}k(x',y')] \\ &+ 2E_x[E_y k(x,y)E_{y'}k(x,y')] - 2(E_{x,y'}k(x,y'))^2 \end{aligned} \tag{10}$$

Proof:

$$\begin{aligned} HSDCC(P_x, P_y, \mathcal{F}) =& \langle \Sigma_{xx} - \Sigma_{yy}, \Sigma_{x'x'} - \Sigma_{y'y'} \rangle_{HS} \\ &= \langle \Sigma_{xx}, \Sigma_{x'x'} \rangle_{HS} + \langle \Sigma_{yy}, \Sigma_{y'y'} \rangle_{HS} - \langle \Sigma_{xx}, \Sigma_{y'y'} \rangle_{HS} - \langle \Sigma_{x'x'}, \Sigma_{yy} \rangle_{HS} \end{aligned} \tag{11}$$

$$
\begin{aligned}
\langle \Sigma_{xx}, \Sigma_{x'x'} \rangle_{HS} =& \langle \widetilde{\Sigma}_{xx} - M_{xx}, \widetilde{\Sigma}_{x'x'} - M_{x'x'} \rangle_{HS} \\
&= \langle \widetilde{\Sigma}_{xx}, \widetilde{\Sigma}_{x'x'} \rangle_{HS} - \langle \widetilde{\Sigma}_{x'x'}, M_{xx} \rangle_{HS} - \langle \widetilde{\Sigma}_{xx}, M_{x'x'} \rangle_{HS} + \langle M_{xx}, M_{x'x'} \rangle_{HS} \\
&= E_{xx'}[\langle \phi(x) \otimes \phi(x), \phi(x') \otimes \phi(x') \rangle_{HS}] - 2E_{x'}[\langle \phi(x') \otimes \phi(x'), \mu_x \otimes \mu_x \rangle_{HS}] \\
&+ \langle \mu_x \otimes \mu_x, \mu_{x'} \otimes \mu_{x'} \rangle_{HS}
\end{aligned} \tag{12}
$$

$$
\begin{aligned}
\langle \Sigma_{yy}, \Sigma_{y'y'} \rangle_{HS} =& E_{yy'}[\langle \phi(y) \otimes \phi(y), \phi(y') \otimes \phi(y') \rangle_{HS}] - 2E_{y'}[\langle \phi(y') \otimes \phi(y'), \mu_y \otimes \mu_y \rangle_{HS}] \\
&+ \langle \mu_y \otimes \mu_y, \mu_{y'} \otimes \mu_{y'} \rangle_{HS}
\end{aligned} \tag{13}
$$

$$
\begin{aligned}
\langle \Sigma_{x'x'}, \Sigma_{yy} \rangle_{HS} = \langle \Sigma_{xx}, \Sigma_{y'y'} \rangle_{HS} =& E_{xy'}[\langle \phi(x) \otimes \phi(x), \phi(y') \otimes \phi(y') \rangle_{HS}] - E_{y'}[\langle \phi(y') \otimes \phi(y'), \mu_x \otimes \mu_x \rangle_{HS}] \\
&- E_x[\langle \phi(x) \otimes \phi(x), \mu_{y'} \otimes \mu_{y'} \rangle_{HS}] + \langle \mu_x \otimes \mu_x, \mu_{y'} \otimes \mu_{y'} \rangle_{HS}
\end{aligned} \tag{14}
$$

We then give the unbiased statistics to $HSDCC(P_x, P_y, \mathcal{F})$ like [2]

2 Figures

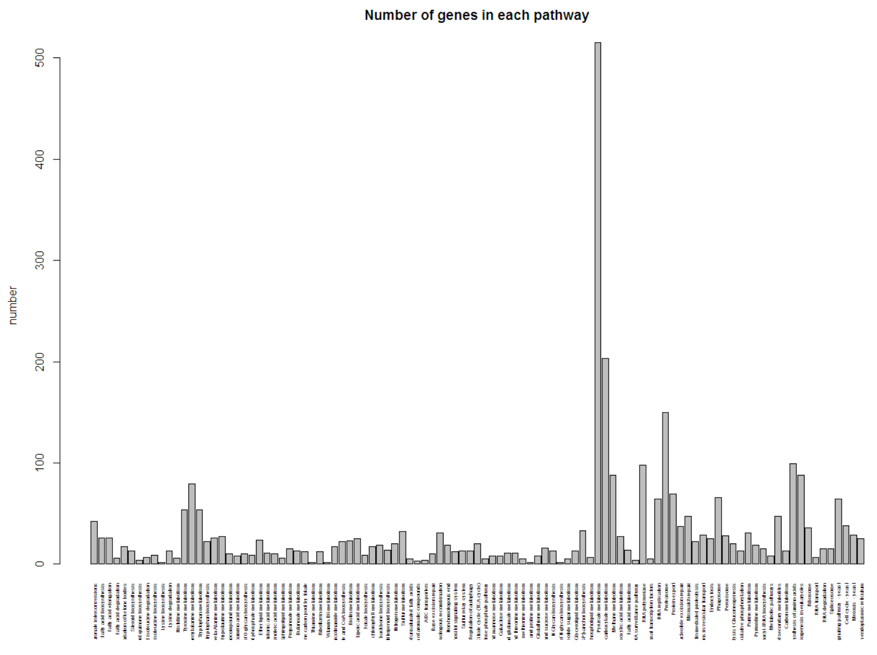

Figure 1 Number of genes in each pathway.

Author details

[1]LMAM, School of Mathematical Sciences, Peking University, Yiheyuan Road, 100871 Beijing, China. [2]Department of Chemical Pathology, Prince of Wales Hospital, Faculty of Medicine, The Chinese University of Hong Kong, Shatin, Hong Kong, China. [3]Center for Quantitative Biology, Peking University, Yiheyuan Road, 100871 Beijing, China. [4]Center for Statistical Sciences, Peking University, Yiheyuan Road, 100871 Beijing, China.

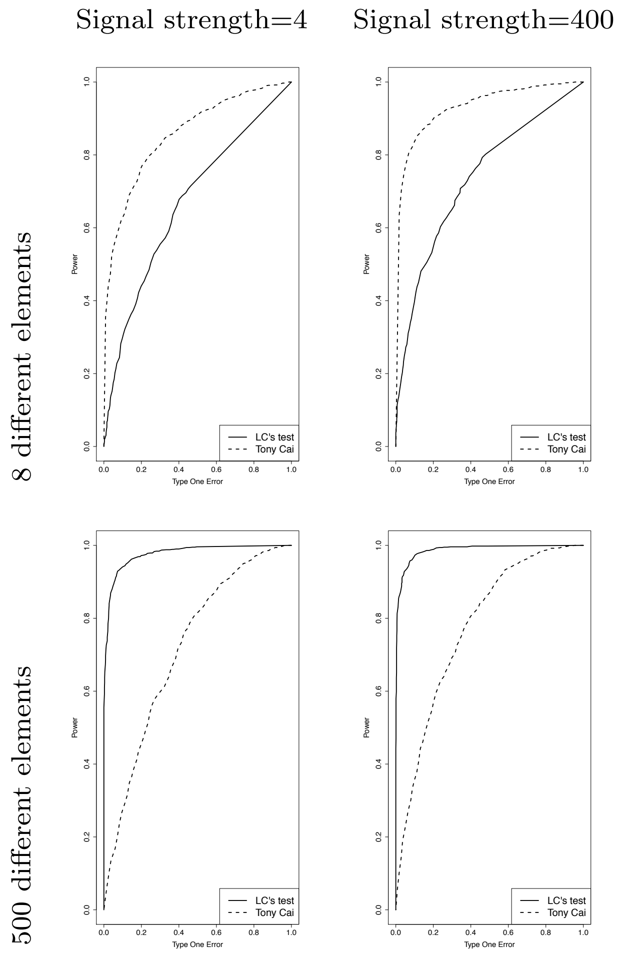

Figure 2 Comparison between Chen's linear method and other method. Number of sample class is 40 and 60, respectively and number of variables is 50. Topleft: The two covariance matrices have eight different elements, each with a magnitude generated from $Unif(0,4) * \max_{1\leq j\leq p} \sigma_{jj}$; Topright: The two covariance matrices have eight different elements, each with a magnitude generated from $Unif(0,400) * \max_{1\leq j\leq p} \sigma_{jj}$; Bottomleft: The two covariance matrices have 500t different elements, each with a magnitude generated from $Unif(0,4) * \max_{1\leq j\leq p} \sigma_{jj}$; Bottomright: The two covariance matrices have 500 different elements, each with a magnitude generated from $Unif(0,400) * \max_{1\leq j\leq p} \sigma_{jj}$;

References

1. Arthur Gretton, Olivier Bousquet, Alex Smola, and Bernhard Schölkopf. Measuring statistical dependence with hilbert-schmidt norms. In *Algorithmic learning theory*, pages 63–77. Springer, 2005.
2. Jun Li, Song Xi Chen, et al. Two sample tests for high-dimensional covariance matrices. *The Annals of Statistics*, 40(2):908–940, 2012.

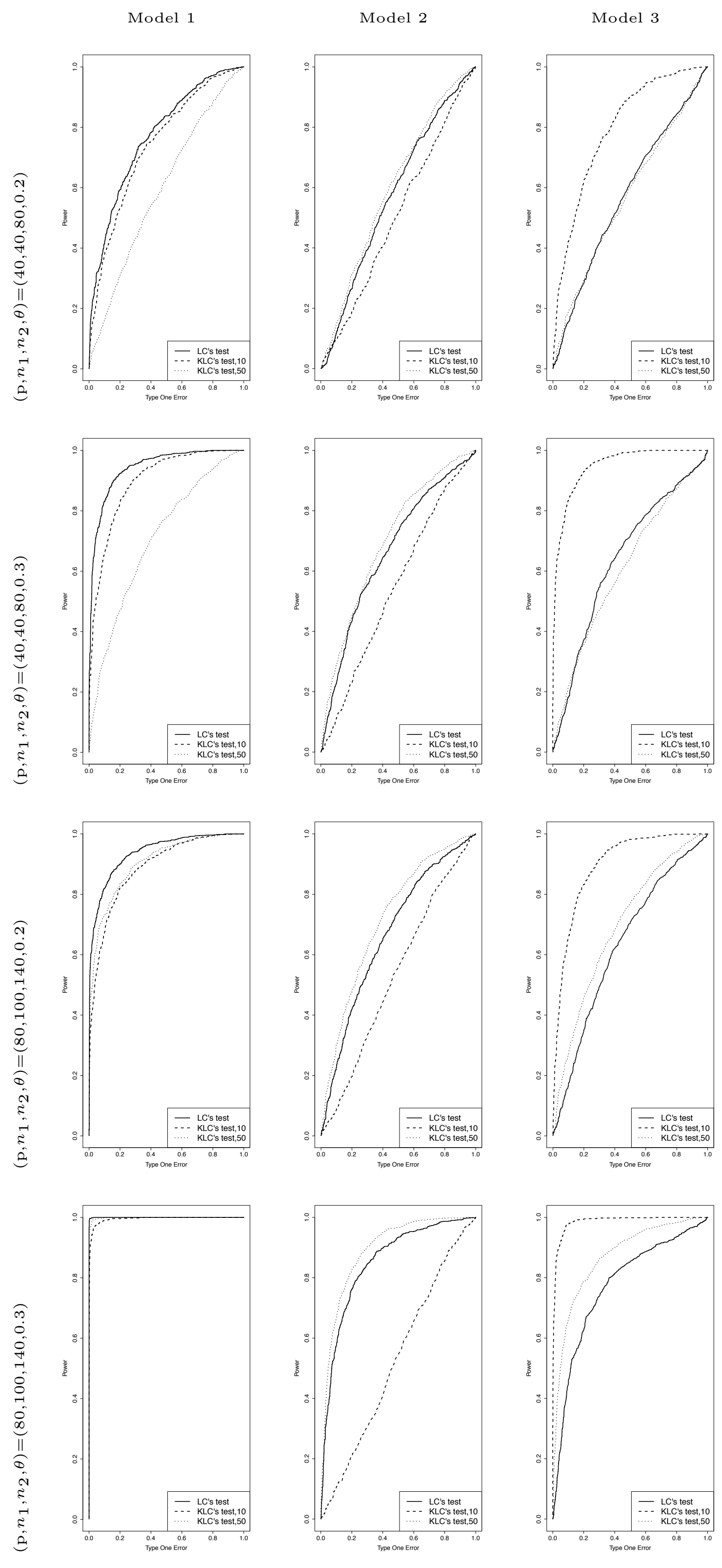

Figure 3 Comparison between linear method and kernel method. Simulations under different setups. Setup of the first column is under model 1, the second column is under model 2 and the third column is under model 3. First row: (p,n_1,n_2,θ)=(40,40,80,0.2); Second row: (p,n_1,n_2,θ)=(40,40,80,0.3); Third row: (p,n_1,n_2,θ)=(80,100,140,0.2); Fourth row: (p,n_1,n_2,θ)=(80,100,140,0.3)
